# Neuroimaging in Alzheimer's Disease for Early Diagnosis: A Comprehensive Review

**DOI:** 10.7759/cureus.38544

**Published:** 2023-05-04

**Authors:** Saikumar Aramadaka, Raam Mannam, Rajagopal Sankara Narayanan, Arpit Bansal, Vishnu R Yanamaladoddi, Sai Suseel Sarvepalli, Shree Laya Vemula

**Affiliations:** 1 Research, Narayana Medical College, Nellore, IND; 2 Research, Narayana Medical College and Hospital, Nellore, IND; 3 Research, ACSR Government Medical College, Nellore, IND

**Keywords:** fdg pet, early diagnosis, tau-protein, amyloid pet, structural mri, functional magnetic resonance imaging, neuroimaging, alzheimer's disease

## Abstract

Alzheimer's disease (AD) is the most common cause of dementia in the elderly, affecting roughly half of those over the age of 85. We briefly discussed the risk factors, epidemiology, and treatment options for AD. The development of therapeutic therapies operating very early in the disease cascade has been spurred by the realization that the disease process begins at least a decade or more before the manifestation of symptoms. Thus, the clinical significance of early diagnosis was emphasized. Using various keywords, a literature search was carried out using PubMed and other databases. For inclusion, pertinent articles were chosen and reviewed. This article has reviewed different neuroimaging techniques that are considered advanced tools to aid in establishing a diagnosis and highlighted the advantages as well as disadvantages of those techniques. Besides, the prevalence of several in vivo biomarkers aided in discriminating affected individuals from healthy controls in the early stages of the disease. Each imaging method has its advantages and disadvantages, hence no single imaging approach can be the optimum modality for diagnosis. This article also commented on a better approach to using these techniques to increase the likelihood of an early diagnosis.

## Introduction and background

Alzheimer's disease (AD), a progressive neurodegenerative disorder, is the most common cause of dementia in older adults, characterized by neurofibrillary tangles (NFTs) and neurotic plaques formed as a result of the accumulation of amyloid-beta (Aβ) peptides, most commonly in the neocortical structures and medial temporal lobe of the brain [1]. In 1907, Aloysius Alzheimer was the first to note these fibrils and plaques in one of the deceased patients, and Emil Kraepelin coined the term AD [2]. It was predicted that by 2050, there will be 152.8 (130.8 to 175.6) million cases of dementia worldwide, up from an expected 57.4 (50.4 to 65.1) million cases in 2019. The expected increases varied geographically, with the biggest increases being seen in eastern sub-Saharan Africa, North Africa, and the Middle East [3]. After the diagnosis of AD dementia, the median survival time was six years in a European memory clinic-based cohort (median 6.2 years [range 6.0-6.5]) [4]. At the age of 85, the prevalence of biologically defined AD is three times higher than that of clinically defined AD, according to a first attempt at estimating prevalence using a biological (rather than clinical) definition [5]. Advanced age is the single most significant risk factor for AD. Symptoms commonly appear after the age of 60 for unexplained reasons. This condition affects around one out of every eight adults aged 65 to 74. Every five years beyond 65, the number of people affected doubles, and AD affects about half of all people over 85. A person's genetic makeup may also raise their chances of developing AD. Specific genes may put a person at a higher risk of AD, which occurs beyond 60, but they do not make it inevitable. People who have one or two copies of the APOE-e4 gene, for example, are more likely to acquire the condition [6]. The Alzheimer's disease continuum describes the course of AD from undetectable brain alterations to brain changes that cause memory issues and eventually physical incapacity. The three stages are preclinical Alzheimer's disease, Alzheimer's-related moderate cognitive impairment (MCI), and Alzheimer's-related dementia [7]. Variable but significant alterations and mild-to-moderate impairments in numerous cognitive, functional, and behavioural domains are seen in people with very mild or mild AD dementia. As evidenced by differential aging and AD impacts on cognitive networks, patterns of change may overlap but are not part of normal cognitive aging [8]. Changes in mood, anxiety, and sleep are some of the first signs that appear years before a clinical diagnosis of dementia. In the preclinical or early phases of AD, anxiety, depressive symptoms, apathy, and withdrawal are all common [9]. Progression to later-stage symptoms like impaired judgment, disorientation, confusion, severe behavioural changes like aggression and agitation, and neuropsychiatric symptoms like delusions and hallucinations can go unnoticed and undertreated until diagnosis [10]. Until 2010, clinical symptom reporting that fit the pattern of memory failure and loss of functional independence in many cognitive domains was used to diagnose and manage AD. The National Institute on Aging-Alzheimer's Association (NIA-AA) and Diagnostic and Statistical Manual of Mental Disorders, Fifth Edition (DSM-5) reclassification systems have expanded the range of AD to encompass pre-clinical illness and MCI, laying the groundwork for the early detection of at-risk patients. There are now a few widely available diagnostic investigations, like body fluids and imaging examinations, that can be used to supplement clinical evaluation for a more reliable diagnosis of AD pathology. The therapy choices for Alzheimer's patients, on the other hand, remain supportive and symptomatic without affecting the long-term prognosis [11].

Considering the disease's increasing prevalence and mortality, the pressure to discover effective procedures for the early identification of AD is immense. Even though no effective medications can reverse existing pathological alterations, delaying disease progression by lowering the risks and early interventions will preserve the patient's functional level for a longer duration. With the realization that immediate action is needed to decrease the burden of AD, a disease with rapidly rising expenses and few treatment choices, the focus has shifted to identifying people far earlier in the disease process. Although there is currently no therapy or cure for dementia, there is an urgent need to increase detection rates so that people at the most significant risk can be identified early and actions are taken to slow or stop the disease's course [12]. Receiving an early diagnosis has several advantages for the patient, including providing a reason for the symptoms and signs they are experiencing and ending their suspicions. Early diagnosis and subsequent access to the appropriate treatments and support can help people gain control of their disease, remain independent in their own homes for extended periods, and maintain a good quality of life for themselves, their families, and their caregivers [13].

The purpose of this article is to review different neuroimaging techniques that are currently available that can detect changes related to AD, highlight the findings related to the disease, evaluate the benefits as well as drawbacks of those techniques, and finally conclude by commenting on better approach using these techniques for early detection.

## Review

This section focuses mainly on imaging techniques that help in the early diagnosis of AD. In the clinical setting, structural neuroimaging is frequently used to differentiate between different kinds of dementia. Computed tomography (CT) and magnetic resonance imaging (MRI) can be used to visualize and assess structural changes in the brain, giving a measure of cerebral atrophy [14]. MRI and CT were the first imaging techniques utilized for Alzheimer's disease. Still, they were employed to rule out other causes of dementia rather than to identify AD at an early stage [15]. Although CT is the most commonly utilized due to its inexpensive cost and ubiquitous availability, MRI provides superior contrast and tissue characterization.

Structural MRI (sMRI)

Protons have rotational momentum polarized in a magnetic field, which is exploited in MRI. This means that a radiofrequency pulse can change the energy state of protons. After the pulse is shut off, the protons will release a radiofrequency signal as they return to their original energy level. "Sequences" can be constructed to be sensitive to various tissue properties by combining different gradients and pulses [15]. Post-mortem investigations have revealed that the medial temporal lobe structures, particularly the entorhinal cortex and hippocampus, are the first to change in AD [16]. Early changes in the medial temporal lobe structures, the entorhinal and perirhinal cortex, and the hippocampus follow a topographical pattern in AD [17]. This pattern aids in diagnosing disease by imaging the above-mentioned brain areas. Studies show a close relation between hippocampal volume loss and AD. Hence the volumetric measure of the hippocampus helps identify the early stages of AD [18-20].

A comparative study done by Barber et al. in 1999 over 104 subjects aged between 75 to 78 years revealed more significant hippocampal atrophy associated with AD than dementia with Lewy bodies (DLB). The above study's findings are supported by another study conducted over 12 months by Mak et al. in 2015 by analysing volumetric changes and spatiotemporal patterns in cortical and subcortical atrophy. Thus, when clinical severity is matched, the severity of hippocampal atrophy in AD is more significant than in DLB and vascular dementia (VaD) [21,22]. As a further advancement in diagnosis, hippocampal subfield measurement came to the surface when Mueller et al. conducted a comparative study in 2010 over 91 subjects comprising AD, MCI, and controls showed a higher sensitivity for hippocampal subfield measurement for early diagnosis, which was backed by serial MRI studies done by Madusanka et al. in 2015 over 90 subjects including similar groups as earlier study [23,24]. 

Apart from volumetric measurement, a cortical thickness examination could be used as a proxy marker for neuronal loss associated with the histological abnormalities in the cortex seen in AD. In 2005, Lerch et al. conducted a clinical trial on 36 subjects (19 AD, 17 controls), demonstrating significant cortical thickness reduction in AD patients [25]. With the advent of sMRI, assessment of both volumes and cortical thickness reduction is possible (Table 1). 

**Table 1:** Summary of studies that used structural MRI for assessment of AD. DLB-Dementia with Lewy Bodies, MCI-Mild Cognitive Impairment, HC-Healthy Controls, MTA-Medial Temporal Atrophy, MTL-Medial Temporal Lobe, VaD-Vascular Dementia, AD-Alzheimer's Disease

REFERENCE	MR measurement	ASSESSMENT	GROUPS	CONCLUSION
Mak et al. [22]	Cortical thickness and subcortical volumes	Atrophy	13 DLB, 23 AD, 33 HC	AD exhibited more significant hippocampal atrophy compared to DLB and HC.
Barber et al. [21]	Medial temporal volume	Atrophy	26 DLB, 28 AD, 24 VaD	AD exhibited more significant hippocampal atrophy compared to DLB and VaD.
Madusanka et al. [24]	Cortical thickness and subcortical volumes	Atrophy	30 AD, 30 HC, 30 MCI	Atrophy of the bilateral CA1, CA2- CA4, and subiculum subfields was higher in the case of AD than in MCI and HC.
Mueller et al. [23]	Cortical thickness and subcortical volumes	Atrophy	18 AD, 20 MCI, 53 HC	subfield measurements might be a more sensitive way to detect MCI and early AD than measurements of the whole hippocampus.
Lerch et al. [25]	Cortical thickness	Atrophy	17 HC, 19 AD	significant cortical thickness decline in AD in temporal, orbitofrontal, and parietal regions, with the most pronounced changes occurring in the allocortical part of the medial temporal lobes, outlining the Para hippocampal gyrus

With roughly 80% accuracy, sMRI alone can predict later conversion to AD in MCI cases [26]. In imaging, hippocampus volumetry is the most well-established structural biomarker for AD, especially for early diagnosis [27]. Studies have also shown volumetric analysis by high-resolution T1 images increases the sensitivity and specificity of diagnosis [28]. Furthermore, as it does not use ionizing radiation, MRI is considered safe and non-invasive. Also, one of the critical advantages of MRI is the availability of equipment in hospitals and research facilities [15].

However, the molecular specificity of sMRI is low. It can't see the histological hallmarks of AD that include Aβ proteins and NFTs. Therefore, it's a step behind molecular pathology [15]. As a result, one of the most significant limitations of sMRI, in general, is the inability to directly detect the effects of Aβ plaques or NFTs in the brain. sMRI demands specialized expertise and can result in high levels of measurement variability [29]. The volume of the hippocampus or medial temporal lobe, the most researched brain region, has low sensitivity and specificity, disqualifying sMRI as a stand-alone add-on test for AD dementia early detection [30]. In atypical symptoms of AD, structural MRI may be unable to detect the disease early as studies show sparing of the hippocampus in atypical AD [31].

Functional MRI (fMRI)

The bold oxygen level-dependent (BOLD) signal, which detects blood flow and volume changes, is used in fMRI to construct dynamic representations of brain activity [32]. Compared to controls, people with AD show little or less activation of the hippocampus and other medial temporal structures during memory tests in a comparative study done by Sperling et al. in 2003. Increased brain activity during encoding in the parietal and posterior cingulate areas suggests that the brain compensates for medial temporal impairment [33]. fMRI can be used to investigate the functional connectivity within specific brain networks during cognitive tasks, typically comparing one condition to a control condition. It can also explore the functional connectivity within particular brain networks during the resting state. The intrinsic oscillations or time course of the BOLD signal between brain regions are investigated using functional connectivity MRI (fc-MRI) techniques [34].

Working memory, visuospatial ability, attention, semantic understanding, and motor performance are areas where fMRI findings in AD have been discovered [35-39]. The at-risk patients performed the fMRI tasks pretty well, which was a common aspect of the studies revealing evidence of elevated fMRI activity. Hyperactivity was seen primarily during successful memory trials in event-related fMRI experiments, suggesting that hyperactivity could be a compensatory strategy in the early stages of AD [40,41].

Functional connections across regions in intrinsic networks implicated in the AD spectrum can be discovered using resting-state fMRI. The default mode network (DMN), which shows more excellent brain activity during rest than task engagement, is one of the networks of interest [42]. The hippocampus and entorhinal cortex and the posterior cingulate cortex (PCC) showed abnormal coactivation during rest in AD according to research done in the USA by Greicies et al. over 26 subjects in 2004. This demonstrates the importance of the MTL in the DMN and confirms connection as a predictor of AD [43]. Zheng et al. published an article on research conducted in 2017 on 70 subjects (32 AD, 38 HC), stressing the disruption of visual, and sensory-motor connections. Evidence of disruption of the dorsal attention network in AD was found through a comparative study by Li et al. in 2012. Research by Zhou et al. on 89 subjects (35 AD, 27 MCI, 27 HC) in 2013 confirmed thalamocortical network disruption. Thus, thalamocortical, dorsal attention, visual, and sensorimotor networks are among the other large-scale brain networks disrupted in Alzheimer's disease [44-46]. Both task-related and resting fMRI techniques can detect early brain damage associated with AD and track therapy response over short periods (Table 2).

**Table 2:** Summary of outcomes of different studies that used functional MRI on Alzheimer's disease subjects. AD-Alzheimer's Disease, MCI-Mild Cognitive Impairment, HC-Healthy Controls, DMN-Default Mode Network

REFERENCE	MODALITY	GROUP	CONCLUSION
Sperling et al. [33]	Task-based fMRI	7 AD, 10 young control subjects, 10 elderly control subjects	During an encoding challenge for Alzheimer's patients, researchers discovered lower hippocampus activity and higher activation in the parietal lobes and posterior cingulate.
Greicius et al. [43]	Resting-State fMRI	15 AD, 18 HC	In Alzheimer's patients, there was less connection between the medial temporal regions and the posterior cingulate cortex.
Zheng et al. [44]	Resting-State fMRI	32 AD, 38 HC	In Alzheimer's patients, functional connectivity was disrupted in numerous significant networks, including the DMN, optical network, and sensorimotor network.
Li et al. [45]	Resting-State fMRI	15 AD, 16 healthy elderly control subjects	Functional connection declines in several regions of the dorsal attention network but not in the ventral attention network
Zhou et al. [46]	Resting-State fMRI	35 AD, 27 MCI, 27 HC	Functional connectivity was reduced in several regions of the thalamocortical network and the thalamo-DMN in Alzheimer's patients. MCI individuals saw similar, although more moderate, declines.

Resting fMRI techniques may be more easily applied to at-risk clinical populations than task fMRI approaches [47]. The morphology of regions with the highest amyloid burden in AD patients overlaps the default network regions demonstrating abnormal task-related fMRI activity and dysconnectivity in MCI and AD [40]. Since no extra equipment is needed, people do not need to be able to execute a cognitive action, and a resting run may be added after safety or volumetric MRI protocol; fc-MRI may be particularly suitable for use in clinical studies [15].

Although fMRI provides unique insight into pathophysiology, it is not recommended for routine clinical usage [48]. The BOLD fMRI response varies between people, and there have been few investigations on the repeatability of fMRI activation in older and cognitively challenged people reported so far [49,50]. Longitudinal fMRI investigations in individuals with progressive dementias are challenging for various reasons. Because these approaches are compassionate to head motion, fMRI is likely to be challenging to use in investigating individuals with more severe cognitive impairment. One of the critical benefits of task fMRI activation investigations is lost if the patients cannot complete the cognitive task correctly. In more seriously handicapped individuals, resting-state fMRI could be more possible [15]. 

Positron emission tomography (PET)

PET is a valuable method for investigating human brain activity in vivo. It can measure brain metabolism, receptor binding for numerous neurotransmitter systems, and changes in regional blood flow without being intrusive. PET radioligands bind to a receptor, transporter, or enzyme. Neuropathology is quantified by the degree of tracer binding or uptake. This technique could be valuable for diagnosing neurological disorders, devising treatments, and monitoring disease development [51]. The brain runs almost entirely on glucose as an energy source. Fluorodeoxyglucose (FDG), a glucose analogue, is a good indicator of brain metabolism and can be detected with PET when labelled with fluorine-18 (half-life 110 minutes) [15]. Regional cerebral glucose consumption (CMRglc) is a precise measurement of integrated local neuronal activity as evaluated by positron emission tomography (PET) and [18F]-2-fluorodeoxyglucose ([^18^F] FDG) [52]. When FDG-PET scans of AD patients were compared to those of healthy people of the same age, patterns of metabolic abnormalities in AD were discovered, leading to the discovery of a so-called FDG-PET endophenotype, which is a characteristic of AD in which specific brain regions or areas are affected in a spatial pattern [15,53]. FDG hypometabolism is a form of endophenotype in Alzheimer's disease, and several studies have found hypometabolism in a set of limbic and related regions [54-57]. When overt dementia is present, FDG-PET has been proven to help confirm the diagnosis of Alzheimer's disease, especially in cases when CSF analysis is uninformative or conflicting [58]. FDG-PET has superior diagnostic value over MR volumetry based on a clinical trial led by Santi et al. [54]. According to Jack and colleagues' hypothetical model of dynamic biomarkers, aberrant FDG-PET precedes alterations apparent with MRI [59]. A deep learning system designed for early AD prediction achieved 82% specificity at 100% sensitivity utilizing FDG-PET of the brain, an average of 75.8 months before the final diagnosis [60]. In extensive research, Silverman et al. employed FDG-PET as a diagnostic tool to distinguish healthy people from people with AD symptoms. FDG-PET has a sensitivity of 94% and a specificity of 73% in detecting AD patients. Furthermore, the sensitivity was 95%, with a specificity of 71% in individuals classified with doubtful or mild dementia [61]. Over time, FDG-PET was found to be a more reliable and specific biomarker for early diagnosis of AD [27,15].

FDG-PET is mainly based on the hypometabolism of regions which in turn suggests neurodegeneration. Therefore, stages of AD before neuronal degeneration are not detected [62]. Any PET scan involves an intravenous injection of a tracer, making it an invasive method, and it is avoided in pregnant and lactating women [63]. Heterogenicity in metabolic patterns between atypical and typical AD may reduce the accuracy of FDG-PET [64]. Aβ plaques and NFTs deposition occur before neurodegeneration initiation, and FDG-PET fails to detect these depositions [65].

Amyloid-PET

Accumulation of Aβ proteins is the earliest pathological change in AD. According to the amyloid cascade hypothesis, loss of regulation between the formation and clearance of Aβ leads to the accumulation of fibrils, further leading to neurodegeneration. Amyloid imaging offers hope for advancement because it enables the direct evaluation of one factor that contributes to cognitive deterioration in AD. The basis of this imaging is grounded on the fact that the hallmark of AD is the histological detection of Aβ plaques at post-mortem autopsy. Currently, there are three FDA-approved amyloid radiotracers used in clinical practice: [11C] Pittsburgh Compound-B ([11C] PiB), [18F] Florbetapir ([18F] FBP), and [18F] Flutemetamol ([18F] FMT) [66-69].

[11C] Pittsburgh Compound-B ([11C] PiB)

Klunk et al. published the first human amyloid PET research utilizing [11C] PiB in January 2004). The half-life of this molecule, which is radiolabelled with 11C, is approximately 20 minutes. As a result, it can only ban on-site cyclotron facilities. Compared to healthy controls, AD patients frequently retained a significant quantity of [11C] PiB in areas of the cerebral cortex that contained substantial amounts of fibrillar Ab plaque deposition in AD patients [70]. Rowe et al. used the distribution volume ratio (DVR) approach to show that AD and DLB patients had more [11C] PiB binding in neocortical areas than healthy control participants but no cortical binding in FTD patients [71]. Rabinovici et al. found that amyloid-PET imaging with PiB might identify Alzheimer's patients from those with other types of dementia like frontotemporal dementia (FTD) [72].

18F-Labeled Radiotracers

[18F] Florbetapir ([18F] FBP) is an 18F-labeled amyloid PET ligand that detects amyloid accumulation in the brain with reasonable accuracy. It is readily taken up through the blood-brain barrier (BBB) and cleansed out of non-amyloid grey matter. It possesses a strong affinity for aggregated Aβ, good separation between the radiotracer amyloid retention and the background signal, and a lengthy, stable pseudo-equilibrium that allows for image capture scheduling flexibility [73,74]. [18F] FBP was the first fluorinated radiotracer, having retention ratios that were substantially linked to PiB [75]. It has a half-life of about 110 minutes, which is longer than [11C] PiB and advantageous as it can be transported from the manufacturing site to a regional PET scanner. [18F] Flutemetamol ([18F] FMT) is a PET radiotracer having a longer half-life than [18F] FBP, allowing it to be dispersed in vivo for a longer time [68]. It is found that as an amyloid imaging agent, [18F] FMT has similar potential as [11C] PiB but with a longer half-life [76].

Cost and availability are the prime obstacles that limit the widespread use of amyloid-PET. However, the issue of availability has been reduced lately by the introduction of 18F-labelled radiotracers. Compared to MRI and FDG-PET, amyloid imaging provides a significantly more binary diagnostic output. While amyloid imaging shows some specificity for AD pathology when that pathology is missing, a negative amyloid-PET scan will be the same regardless of dementia's non-AD cause. In contrast, when an amyloid-PET scan is ambiguously negative in both circumstances, MRI and FDG-PET may indicate a frontotemporal or vascular disease [15]. [11C] PiB scans showed the amyloid burden in elderly patients without any cognitive deficit but with one or more alleles of the APOE4 gene and uncontrolled hypertension [77]. Clinical, mental, and cerebrospinal fluid (CSF) indicators associated with Alzheimer's disease may be detected before cerebral Aβ plaques are detected with amyloid imaging agents like PiB, which predominantly identify fibrillar Aβ plaques. A study by Cairns et al. showed that amyloid deposition had been found histologically even before in vivo signals become positive [78].

Tau-PET

There has been no effective reversal or reduction of AD symptoms by targeting Aβ. As a result, the focus of AD treatment research has switched to the function of tau in AD pathogenesis. Even though tau pathology is thought to be secondary to Aβ accumulation, tau pathological alterations are more closely linked to disease progression and cognitive impairments [79]. Tau is a protein abundantly expressed in CNS associated with neurons' microtubules and helps stabilize the microtubules of axons [80]. Tau protein forms insoluble fibers called paired helical filaments (PHFs) after hyperphosphorylation in AD, which later form aggregates in the cytoplasm of neurons leading to the formation of NFTs [81,82]. The NFT load has been linked to the severity of dementia and neurodegeneration in postmortem investigations rather than plaques [83]. Tau aggregation is thought to play a significant role in neurodegeneration observed in AD despite knowledge of the proper mechanism [84]. NFTs begin in the trans-entorhinal region, then spread to the entorhinal cortex and hippocampus, eventually affecting the temporal cortex and other cortical areas [16]. Radiotracers developed for Tau-PET must first cross the BBB and should reach NFTs located intracellularly.

Additionally, they must exhibit high specificity and rapid clearance rate [85]. The ultrastructural PHF type of tau aggregates are most prevalent in AD. Hence most attempts to produce tau-PET tracers have focused on imaging these PHFs [86]. A few of the tracers used in tau-PET are pyrido−indole derivatives, quinoline derivatives, PBB3, and 18F-FDDNP.

Pyrido-Indole Derivatives

Flortaucipir and 18F−T808 are fluorinated pyrido-indole derivatives exhibiting a high affinity to tau compared to amyloid plaques [87,88]. Flortaucipir shows a relationship to multiple binding sites on tau fibril [89]. With modest levels of white matter uptake, flortaucipir exhibited over a 25-fold preference for tau against A plaques [88]. However, flortaucipir had a poor affinity for tau aggregates, mostly of straight tau filaments, indicating that it would not be a good radiotracer for disorders other than Alzheimer's [65]. 18FT808 had a high tau affinity, quick absorption, and clearance, but it had the drawback of defluorination followed by bone uptake [90].

18F-FDDNP

The PET radiotracer 18F-FDDNP was the first to be used in clinical PET imaging of tau pathology in Alzheimer's patients, which stains neurofibrillary tangles and senile plaques prion plaques, and cerebral amyloid angiopathy [91,92]. 18F-FDDNP binds diffusely throughout the neocortex and hippocampus in vitro autoradiography of AD brain slices, demonstrating that 18F-FDDNP binds to both amyloid plaques and neurofibrillary tangles [93,94]. 18F-FDDNP prefers to attach to Aβ rather than tau [95,86].

PBB3

PBB3 is an 11C-labelled radiotracer that can detect AD and non-AD tauopathies [95]. It has an affinity to the binding site on tau that is different from other radiotracers [89]. This compound binds to a broad spectrum of tau isoforms and has a 50-fold greater binding affinity for tau than Aβ [96].

Quinoline Derivatives

The earliest selective tau PET tracers were based on quinoline and benzimidazole derivatives, and they were designed to image PHF tau. In their investigation, Okamura et al. created three novel compounds, BF-126, BF-158, and BF-170, as potential probes for in vivo tau-PET imaging in the brain. The chemicals have a high absorption rate and elimination rate from brain tissue. Additionally, these compounds could see NFTs and PHF-type neuritis in a neuropathological examination, indicating that quinoline and benzimidazole derivatives might be viable tau-PET tracers [97]. 18F-THK523 was the first 18F-labeled arylquinoline derivative developed as a radiotracer for tau imaging. Compared to amyloid (1-42) fibrils, it binds to a more significant number of binding sites on recombinant tau (K18280K) and its excellent affinity and selectivity for tau pathology in vitro and in vivo have been proven in preclinical studies [98]. THK-5105 and THK-5117 demonstrated a superior binding relationship to tau-rich AD brain homogenates and tau protein aggregates compared to THK-523 on performing In-vitro binding assays [99]. Later, (18)F-THK5351 was developed with lower retention in subcortical white matter due to its rapid dissociation than THK5117. It showcased higher contrast and fast kinetics as well [100].

Compared to Aβ, tau-PET tracers are still in development, and clinical confirmation of the tracers is necessary [101]. The creation of new tau tracers is a continuous process in which numerous pharmaceutical firms are working to enhance the tracers' pharmacokinetics and pharmacodynamics [84].

However, every technique discussed above has its limitations and advantages, rendering us in a difficult position to select a single modality as an epitome for the effective early diagnosis of AD (Table 3).

**Table 3:** Summary of advantages and limitations of different neuroimaging techniques in Alzheimer's disease. AD-Alzheimer's Disease, MCI-Mild Cognitive Impairment, MRI-Magnetic Resonance Imaging, NFTs-Neurofibrillary tangles, Aβ-Amyloid beta, PiB-Pittsburgh Compound B, FDG-Fluorodeoxyglucose, PET-Positron Emission Tomography, PHF-Paired Helical Filament

IMAGING TECHNIQUE	ADVANTAGES	LIMITATIONS
Structural MRI	1) MRI scanners are widely available and safe. 2) Good in volume prediction. 3) Atrophy correlates with cognitive decline.	1) Reduced hippocampal volume is not specific to AD. 2) NFTs and Aβ plaques cannot be observed directly. 3} Absence of constant atrophic patterns in AD subtypes. 4) Atypical AD is difficult to find.
Functional MRI	1) evaluation of brain function non-invasively, safely, and effectively. 2) Areas of abnormal activity correlate with areas of amyloid deposition. 3) Functional connectivity between brain networks can be investigated.	1) Fails to detect Aβ plaques and NFTs. 2) Highly sensitive to head motion. 3) Expensive. 4) Relatively low temporal resolution.
FDG-PET	1) Has high sensitivity and specificity. 2) Metabolic pattern differences help predict the risk for conversion to AD. Diminished FDG uptake precedes clinical manifestation. 3) Difference in topographic progression aids in finding AD variants. 4) Considerable research yielded an FDG-PET endophenotype that can be utilized for comparison.	1) Availability and cost are definite issues. 2) Injection of radiolabelled tracer render it to be invasive. 3) Glucose hypometabolism is not specific to AD.
Amyloid-PET	1) Visualisation of Aβ plaques is possible and is considered the hallmark of early AD. 2) PiB's retention time predicts MCI conversion to AD.	1) Weak association between Aβ deposition and illness severity. 2) Injection of radiolabelled tracer render it to be invasive. 3) Some of the healthy controls also show PiB uptake. 4) Aβ buildup in atypical forms is poorly understood.
Tau-PET	1) Tau buildup is believed to be closely associated with cognitive impairment. 2) high affinity of radiotracers for PHF tau. 3) Strong correlation between neurodegeneration and NFTs.	1) Injection of radiolabelled tracer render it to be invasive. 2) High degree of variability in tau morphology amongst AD subtypes. 3) Still-new area of study.

## Conclusions

Alzheimer's mainly affects older adults causing dementia due to neurodegeneration. AD is a disease with a long period of subclinical stages. The role of imaging techniques for early diagnosis is mostly utilized in prevention studies. This article discussed imaging modalities commonly used to diagnose AD, their advantages, and their limitations. However, advanced MRI techniques like arterial spin labeling and diffuse tensor imaging (DTI) are also pitched to understand AD. In PET imaging, efforts to develop suitable ligands with high selectivity and affinity are ongoing. Therefore, no single imaging technique is sufficient for early diagnosis, which led to a new concept called ''multi-modal imaging''. In this method, to increase diagnostic accuracy, the same patient is subjected to multiple techniques. By this, the strengths and weaknesses of different techniques complement each other increasing the diagnostic value. Among the techniques mentioned above, PET imaging is obviously to be relatively more helpful in early diagnosis. But in a practical world, all suspected cases cannot be subjected to PET imaging. The clinical significance of this article is to emphasize the fact that no radio diagnostic method is conclusive on its own and multi-modal imaging helps narrow down the cases in definite need of advanced molecular imaging, therefore avoiding suspected cases from going through unnecessary imaging. This article should reinforce the minds of practitioners that advanced, invasive techniques are not the sole modalities for early diagnosis. However, we recommend further studies should be carried out to develop more sophisticated techniques and discover better, safe, accessible, and cost-effective biomarkers to increase our current probability to diagnose AD early.

## References

[1] De-Paula VJ, Radanovic M, Diniz BS, Forlenza OV (2012). Alzheimer's disease. Subcell Biochem.

[2] Bondi MW, Edmonds EC, Salmon DP (2017). Alzheimer's disease: past, present, and future. J Int Neuropsychol Soc.

[3] (2022). Estimation of the global prevalence of dementia in 2019 and forecasted prevalence in 2050: an analysis for the Global Burden of Disease Study 2019. Lancet Public Health.

[4] Rhodius-Meester HF, Tijms BM, Lemstra AW (2019). Survival in memory clinic cohort is short, even in young-onset dementia. J Neurol Neurosurg Psychiatry.

[5] Jack CR Jr, Therneau TM, Weigand SD (2019). Prevalence of biologically vs clinically defined Alzheimer spectrum entities using the National Institute on Aging-Alzheimer’s Association research framework. JAMA Neurol.

[6] Ballard C, Gauthier S, Corbett A, Brayne C, Aarsland D, Jones E (2011). Alzheimer's disease. Lancet.

[7] Jack CR Jr, Bennett DA, Blennow K (2018). Toward a biological definition of Alzheimer's disease. Alzheimers Dement.

[8] Chhatwal JP, Schultz AP, Johnson KA (2018). Preferential degradation of cognitive networks differentiates Alzheimer's disease from ageing. Brain.

[9] Jost BC, Grossberg GT (1996). The evolution of psychiatric symptoms in Alzheimer's disease: a natural history study. J Am Geriatr Soc.

[10] Atri A (2019). The Alzheimer's disease clinical spectrum: diagnosis and management. Med Clin North Am.

[11] Weller J, Budson A (2018). Current understanding of Alzheimer's disease diagnosis and treatment. F1000Res.

[12] Rasmussen J, Langerman H (2019). Alzheimer's disease - why we need early diagnosis. Degener Neurol Neuromuscul Dis.

[13] (2015). Why early diagnosis of dementia is important. https://www.scie.org.uk/dementia/symptoms/diagnosis/early-diagnosis.asp.

[14] Kemp PM, Holmes C (2007). Imaging in dementia with Lewy bodies: a review. Nucl Med Commun.

[15] Johnson KA, Fox NC, Sperling RA, Klunk WE (2012). Brain imaging in Alzheimer disease. Cold Spring Harb Perspect Med.

[16] Braak H, Braak E (1991). Neuropathological stageing of Alzheimer-related changes. Acta Neuropathol.

[17] Mosconi L (2013). Glucose metabolism in normal aging and Alzheimer's disease: methodological and physiological considerations for PET studies. Clin Transl Imaging.

[18] Jack CR Jr, Dickson DW, Parisi JE (2002). Antemortem MRI findings correlate with hippocampal neuropathology in typical aging and dementia. Neurology.

[19] Gosche KM, Mortimer JA, Smith CD, Markesbery WR, Snowdon DA (2002). Hippocampal volume as an index of Alzheimer neuropathology: findings from the Nun Study. Neurology.

[20] Bobinski M, de Leon MJ, Wegiel J (2000). The histological validation of post-mortem magnetic resonance imaging-determined hippocampal volume in Alzheimer's disease. Neuroscience.

[21] Barber R, Gholkar A, Scheltens P, Ballard C, McKeith IG, O'Brien JT (1999). Medial temporal lobe atrophy on MRI in dementia with Lewy bodies. Neurology.

[22] Mak E, Su L, Williams GB, Watson R, Firbank MJ, Blamire AM, O'Brien JT (2015). Progressive cortical thinning and subcortical atrophy in dementia with Lewy bodies and Alzheimer's disease. Neurobiol Aging.

[23] Mueller SG, Schuff N, Yaffe K, Madison C, Miller B, Weiner MW (2010). Hippocampal atrophy patterns in mild cognitive impairment and Alzheimer's disease. Hum Brain Mapp.

[24] Madusanka N, Choi HK, So JH, Choi BK, Park HG (2019). One-year follow-up study of hippocampal subfield atrophy in Alzheimer’s disease and normal aging. Curr Med Imaging Rev.

[25] Lerch JP, Pruessner JC, Zijdenbos A, Hampel H, Teipel SJ, Evans AC (2005). Focal decline of cortical thickness in Alzheimer's disease identified by computational neuroanatomy. Cereb Cortex.

[26] Ritchie C, Smailagic N, Noel-Storr AH, Takwoingi Y, Flicker L, Mason SE, McShane R (2014). Plasma and cerebrospinal fluid amyloid beta for the diagnosis of Alzheimer's disease dementia and other dementias in people with mild cognitive impairment (MCI). Cochrane Database Syst Rev.

[27] Henriques AD, Benedet AL, Camargos EF, Rosa-Neto P, Nóbrega OT (2018). Fluid and imaging biomarkers for Alzheimer's disease: where we stand and where to head to. Exp Gerontol.

[28] Saad SS, Alashwah MM, Alsafa AA (2020). The role of brain structural magnetic resonance imaging in the assessment of hippocampal subfields in Alzheimer’s disease. Egypt J Radiol Nucl Med 51.

[29] Cash DM, Rohrer JD, Ryan NS, Ourselin S, Fox NC (2014). Imaging endpoints for clinical trials in Alzheimer's disease. Alzheimers Res Ther.

[30] Lombardi G, Crescioli G, Cavedo E (2020). Structural magnetic resonance imaging for the early diagnosis of dementia due to Alzheimer's disease in people with mild cognitive impairment. Cochrane Database Syst Rev.

[31] Firth NC, Primativo S, Marinescu RV (2019). Longitudinal neuroanatomical and cognitive progression of posterior cortical atrophy. Brain.

[32] Logothetis NK, Pauls J, Augath M, Trinath T, Oeltermann A (2001). Neurophysiological investigation of the basis of the fMRI signal. Nature.

[33] Sperling RA, Bates JF, Chua EF (2003). fMRI studies of associative encoding in young and elderly controls and mild Alzheimer's disease. J Neurol Neurosurg Psychiatry.

[34] Fox MD, Snyder AZ, Vincent JL, Corbetta M, Van Essen DC, Raichle ME (2005). The human brain is intrinsically organized into dynamic, anticorrelated functional networks. Proc Natl Acad Sci U S A.

[35] McGeown WJ, Shanks MF, Forbes-McKay KE, Venneri A (2009). Patterns of brain activity during a semantic task differentiate normal aging from early Alzheimer's disease. Psychiatry Res.

[36] Yetkin FZ, Rosenberg RN, Weiner MF, Purdy PD, Cullum CM (2006). FMRI of working memory in patients with mild cognitive impairment and probable Alzheimer's disease. Eur Radiol.

[37] Vidoni ED, Thomas GP, Honea RA, Loskutova N, Burns JM (2012). Evidence of altered corticomotor system connectivity in early-stage Alzheimer's disease. J Neurol Phys Ther.

[38] Thiyagesh SN, Farrow TF, Parks RW (2009). The neural basis of visuospatial perception in Alzheimer's disease and healthy elderly comparison subjects: an fMRI study. Psychiatry Res.

[39] Li C, Zheng J, Wang J, Gui L, Li C (2009). An fMRI stroop task study of prefrontal cortical function in normal aging, mild cognitive impairment, and Alzheimer's disease. Curr Alzheimer Res.

[40] Sperling RA, Laviolette PS, O'Keefe K (2009). Amyloid deposition is associated with impaired default network function in older persons without dementia. Neuron.

[41] Dickerson BC, Sperling RA (2008). Functional abnormalities of the medial temporal lobe memory system in mild cognitive impairment and Alzheimer's disease: insights from functional MRI studies. Neuropsychologia.

[42] Greicius MD, Krasnow B, Reiss AL, Menon V (2003). Functional connectivity in the resting brain: a network analysis of the default mode hypothesis. Proc Natl Acad Sci U S A.

[43] Greicius MD, Srivastava G, Reiss AL, Menon V (2004). Default-mode network activity distinguishes Alzheimer's disease from healthy aging: evidence from functional MRI. Proc Natl Acad Sci U S A.

[44] Zheng W, Liu X, Song H, Li K, Wang Z (2017). Altered functional connectivity of cognitive-related cerebellar subregions in Alzheimer’s disease. Front Aging Neurosci.

[45] Li R, Wu X, Fleisher AS, Reiman EM, Chen K, Yao L (2012). Attention-related networks in Alzheimer's disease: a resting functional MRI study. Hum Brain Mapp.

[46] Zhou B, Liu Y, Zhang Z (2013). Impaired functional connectivity of the thalamus in Alzheimer's disease and mild cognitive impairment: a resting-state fMRI study. Curr Alzheimer Res.

[47] Fleisher AS, Sherzai A, Taylor C, Langbaum JB, Chen K, Buxton RB (2009). Resting-state BOLD networks versus task-associated functional MRI for distinguishing Alzheimer's disease risk groups. Neuroimage.

[48] Rocchi L, Niccolini F, Politis M (2015). Recent imaging advances in neurology. J Neurol.

[49] Clément F, Belleville S (2009). Test-retest reliability of fMRI verbal episodic memory paradigms in healthy older adults and in persons with mild cognitive impairment. Hum Brain Mapp.

[50] Putcha D, O'Keefe K, LaViolette P (2011). Reliability of functional magnetic resonance imaging associative encoding memory paradigms in non-demented elderly adults. Hum Brain Mapp.

[51] Politis M, Piccini P (2012). Positron emission tomography imaging in neurological disorders. J Neurol.

[52] Rocher AB, Chapon F, Blaizot X, Baron JC, Chavoix C (2003). Resting-state brain glucose utilization as measured by PET is directly related to regional synaptophysin levels: a study in baboons. Neuroimage.

[53] Liu X, Chen K, Wu T, Weidman D, Lure F, Li J (2018). Use of multimodality imaging and artificial intelligence for diagnosis and prognosis of early stages of Alzheimer's disease. Transl Res.

[54] De Santi S, de Leon MJ, Rusinek H (2001). Hippocampal formation glucose metabolism and volume losses in MCI and AD. Neurobiol Aging.

[55] Minoshima S, Giordani B, Berent S, Frey KA, Foster NL, Kuhl DE (1997). Metabolic reduction in the posterior cingulate cortex in very early Alzheimer's disease. Ann Neurol.

[56] Foster NL, Chase TN, Fedio P, Patronas NJ, Brooks RA, Di Chiro G (1983). Alzheimer's disease: focal cortical changes shown by positron emission tomography. Neurology.

[57] Reiman EM, Caselli RJ, Yun LS (1996). Preclinical evidence of Alzheimer's disease in persons homozygous for the epsilon 4 allele for apolipoprotein E. N Engl J Med.

[58] Perini G, Rodriguez-Vieitez E, Kadir A, Sala A, Savitcheva I, Nordberg A (2021). Clinical impact of (18)F-FDG-PET among memory clinic patients with uncertain diagnosis. Eur J Nucl Med Mol Imaging.

[59] Jack CR Jr, Knopman DS, Jagust WJ (2010). Hypothetical model of dynamic biomarkers of the Alzheimer's pathological cascade. Lancet Neurol.

[60] Ding Y, Sohn JH, Kawczynski MG (2019). A deep learning model to predict a diagnosis of Alzheimer disease by using (18)F-FDG PET of the brain. Radiology.

[61] Silverman DH, Small GW, Chang CY (2001). Positron emission tomography in evaluation of dementia: regional brain metabolism and long-term outcome. JAMA.

[62] Drzezga A, Altomare D, Festari C (2018). Diagnostic utility of 18F-Fluorodeoxyglucose positron emission tomography (FDG-PET) in asymptomatic subjects at increased risk for Alzheimer's disease. Eur J Nucl Med Mol Imaging.

[63] Kapoor M, Kasi A (2022). PET Scanning. https://www.ncbi.nlm.nih.gov/books/NBK559089/.

[64] Vanhoutte M, Lopes R, Maureille A (2016). P1- 291: hypometabolism patterns using FDG-PET in typical and atypical sporadic forms of early-onset Alzheimer’s disease. Alzheimer’s Dement.

[65] van Oostveen WM, de Lange EC (2021). Imaging techniques in Alzheimer's disease: a review of applications in early diagnosis and longitudinal monitoring. Int J Mol Sci.

[66] Counts SE, Ikonomovic MD, Mercado N, Vega IE, Mufson EJ (2017). Biomarkers for the early detection and progression of Alzheimer's disease. Neurotherapeutics.

[67] Hardy JA, Higgins GA (1992). Alzheimer's disease: the amyloid cascade hypothesis. Science.

[68] Vandenberghe R, Adamczuk K, Dupont P, Laere KV, Chételat G (2013). Amyloid PET in clinical practice: its place in the multidimensional space of Alzheimer's disease. Neuroimage Clin.

[69] Suppiah S, Didier MA, Vinjamuri S (2019). The who, when, why, and how of PET amyloid imaging in management of Alzheimer's disease-review of literature and interesting images. Diagnostics (Basel).

[70] Klunk WE, Engler H, Nordberg A (2004). Imaging brain amyloid in Alzheimer's disease with Pittsburgh Compound-B. Ann Neurol.

[71] Rowe CC, Ng S, Ackermann U (2007). Imaging beta-amyloid burden in aging and dementia. Neurology.

[72] Rabinovici GD, Furst AJ, O'Neil JP (2007). 11C-PiB PET imaging in Alzheimer disease and frontotemporal lobar degeneration. Neurology.

[73] Clark CM, Schneider JA, Bedell BJ (2011). Use of florbetapir-PET for imaging beta-amyloid pathology. JAMA.

[74] Wong DF, Rosenberg PB, Zhou Y (2010). In vivo imaging of amyloid deposition in Alzheimer disease using the radioligand 18F-AV-45 (florbetapir [corrected] F 18). J Nucl Med.

[75] Wolk DA, Zhang Z, Boudhar S, Clark CM, Pontecorvo MJ, Arnold SE (2012). Amyloid imaging in Alzheimer's disease: comparison of florbetapir and Pittsburgh compound-B positron emission tomography. J Neurol Neurosurg Psychiatry.

[76] Ataka S, Takeda A, Mino T (2014). Comparison of [18F] flutemetamol and [11C] PiB PET images. Alzheimer’s Dement.

[77] Rodrigue KM, Rieck JR, Kennedy KM, Devous MD Sr, Diaz-Arrastia R, Park DC (2013). Risk factors for β-amyloid deposition in healthy aging: vascular and genetic effects. JAMA Neurol.

[78] Cairns NJ, Ikonomovic MD, Benzinger T (2009). Absence of Pittsburgh compound B detection of cerebral amyloid beta in a patient with clinical, cognitive, and cerebrospinal fluid markers of Alzheimer disease: a case report. Arch Neurol.

[79] Agadjanyan MG, Zagorski K, Petrushina I (2017). Humanized monoclonal antibody armanezumab specific to N-terminus of pathological tau: characterization and therapeutic potency. Mol Neurodegener.

[80] Goedert M, Crowther RA, Garner CC (1991). Molecular characterization of microtubule-associated proteins tau and MAP2. Trends Neurosci.

[81] Grundke-Iqbal I, Iqbal K, Quinlan M, Tung YC, Zaidi MS, Wisniewski HM (1986). Microtubule-associated protein tau. A component of Alzheimer's paired helical filaments. J Biol Chem.

[82] Braak H, Braak E (1997). Frequency of stages of Alzheimer-related lesions in different age categories. Neurobiol Aging.

[83] Arriagada PV, Growdon JH, Hedley-Whyte ET, Hyman BT (1992). Neurofibrillary tangles but not senile plaques parallel duration and severity of Alzheimer's disease. Neurology.

[84] Okamura N, Harada R, Ishiki A, Kikuchi A, Nakamura T, Kudo Y (2018). The development and validation of tau PET tracers: current status and future directions. Clin Transl Imaging.

[85] Villemagne VL, Fodero-Tavoletti MT, Masters CL, Rowe CC (2015). Tau imaging: early progress and future directions. Lancet Neurol.

[86] Villemagne VL, Okamura N (2014). In vivo tau imaging: obstacles and progress. Alzheimers Dement.

[87] Bao W, Jia H, Finnema S, Cai Z, Carson RE, Huang YH (2017). PET imaging for early detection of Alzheimer's disease: from pathologic to physiologic biomarkers. PET Clin.

[88] Valotassiou V, Malamitsi J, Papatriantafyllou J (2018). SPECT and PET imaging in Alzheimer's disease. Ann Nucl Med.

[89] Murugan NA, Nordberg A, Ågren H (2018). Different positron emission tomography tau tracers bind to multiple binding sites on the tau fibril: insight from computational modeling. ACS Chem Neurosci.

[90] Chien DT, Bahri S, Szardenings AK (2013). Early clinical PET imaging results with the novel PHF-tau radioligand [F-18]-T807. J Alzheimers Dis.

[91] Harada R, Okamura N, Furumoto S, Tago T, Yanai K, Arai H, Kudo Y (2016). Characteristics of tau and its ligands in PET imaging. Biomolecules.

[92] Bresjanac M, Smid LM, Vovko TD, Petric A, Barrio JR, Popovic M (2003). Molecular-imaging probe 2-(1-[6-[(2-fluoroethyl)(methyl) amino]-2-naphthyl]ethylidene) malononitrile labels prion plaques in vitro. J Neurosci.

[93] Agdeppa ED, Kepe V, Liu J (2001). Binding characteristics of radiofluorinated 6-dialkylamino-2-naphthylethylidene derivatives as positron emission tomography imaging probes for beta-amyloid plaques in Alzheimer's disease. J Neurosci.

[94] Agdeppa ED, Kepe V, Petri A (2003). In vitro detection of (S)-naproxen and ibuprofen binding to plaques in the Alzheimer's brain using the positron emission tomography molecular imaging probe 2-(1-[6-[(2-[(18)F]fluoroethyl)(methyl)amino]-2-naphthyl]ethylidene)malononitrile. Neuroscience.

[95] Maruyama M, Shimada H, Suhara T (2013). Imaging of tau pathology in a tauopathy mouse model and in Alzheimer patients compared to normal controls. Neuron.

[96] Kimura Y, Ichise M, Ito H (2015). PET quantification of tau pathology in human brain with 11C-PBB3. J Nucl Med.

[97] Okamura N, Suemoto T, Furumoto S (2005). Quinoline and benzimidazole derivatives: candidate probes for in vivo imaging of tau pathology in Alzheimer's disease. J Neurosci.

[98] Fodero-Tavoletti MT, Okamura N, Furumoto S (2011). 18F-THK523: a novel in vivo tau imaging ligand for Alzheimer's disease. Brain.

[99] Okamura N, Furumoto S, Harada R (2013). Novel 18F-labeled arylquinoline derivatives for noninvasive imaging of tau pathology in Alzheimer disease. J Nucl Med.

[100] Harada R, Okamura N, Furumoto S (2016). 18F-THK5351: a novel PET radiotracer for imaging neurofibrillary pathology in Alzheimer disease. J Nucl Med.

[101] Frisoni GB, Boccardi M, Barkhof F (2017). Strategic roadmap for an early diagnosis of Alzheimer's disease based on biomarkers. Lancet Neurol.

